# Modelling the Transference of Paediatric Patients with Inborn Errors of Metabolism to Adult Hospitals: Clinical Experience

**DOI:** 10.3390/jcm15010081

**Published:** 2025-12-22

**Authors:** Aida Deudero, Esther Lasheras, Roser Ventura, Cristina Montserrat-Carbonell, José César Milisenda, Natalia Juliá-Palacios, Ana Matas, María de Talló Forga-Visa, Rosa María López-Galera, Judit García-Villoria, Mercè Placeres, Adriana Pané, Glòria Garrabou, Antonia Ribes, Francesc Cardellach, Pedro Juan Moreno-Lozano, Àngels Garcia-Cazorla, Jaume Campistol, IEM-SJD-HCB Consortia

**Affiliations:** 1 Inherited Metabolic Diseases and Muscular Disorders’ Research Laboratory, Centre de Recerca Biomèdica CELLEX, Institut d’Investigacions Biomèdiques August Pi i Sunyer (IDIBAPS), Faculty of Medicine and Health Sciences, University of Barcelona, 08036 Barcelona, Spainjcmilise@clinic.cat (J.C.M.); anmatas@clinic.cat (A.M.); pjmoreno@clinic.cat (P.J.M.-L.); 2 Transition Area, Quality Department, Hospital Sant Joan de Déu, 08950 Barcelona, Spain; esther.lasheras@sjd.es (E.L.); nataliaalexandra.julia@sjd.es (N.J.-P.); angeles.garcia@sjd.es (À.G.-C.); jaime.campistol@sjd.es (J.C.); 3 Internal Medicine Department, Hospital Clinic of Barcelona, 08036 Barcelona, Spain; ventura@clinic.cat; 4 Endocrinology Department, Hospital Clinic of Barcelona, 08036 Barcelona, Spain; cmontse@clinic.cat (C.M.-C.); mtforga@clinic.cat (M.d.T.F.-V.); pane@clinic.cat (A.P.); 5 Centro de Investigación Biomédica en Red de Enfermedades Raras (CIBERER), Instituto de Salud Carlos III (ISCIII), 28029 Madrid, Spain; rmlopez@clinic.cat (R.M.L.-G.); jugarcia@clinic.cat (J.G.-V.); aribes@clinic.cat (A.R.); 6 Metabolic Diseases Unit, Department of Neurology, Hospital Sant Joan de Déu and MetabERN, 08950 Barcelona, Spain; 7 Division of Inborn Errors of Metabolism-IBC, Biochemical and Molecular Genetics Department, Hospital Clinic of Barcelona, IDIBAPS, 08036 Barcelona, Spain; 8 Pharmacy Department, Hospital Clinic of Barcelona, 08036 Barcelona, Spain; placeres@clinic.cat

**Keywords:** inborn errors of metabolism, transition process, transference model, rare diseases, quality of care, interdisciplinary team

## Abstract

**Background/Objectives**: Inborn errors of metabolism (IEM) are chronic, life-threatening genetic disorders with a significant cumulative prevalence worldwide. Advances in early diagnosis and treatment have significantly increased life expectancy, underscoring the need for specialised adult care units and the establishment of structured transition programmes from paediatric to adult services. We hereby present a functional transition model for IEM patients and share our implementation experience. **Methods**: Initiated in 2012, the partnership between the paediatric Hospital Sant Joan de Déu (HSJD) and the adult-care centre at Hospital Clinic of Barcelona (HCB) culminated in 2019 with the transference of the first IEM patients under the structured A10! Programme. This model is structured around the transition units of paediatric and adult centres to guarantee communication and functional management. Regular monthly meetings at each centre and joint quarterly sessions allowed for protocol harmonisation and personalised care planning. Coordinated engagement of the multidisciplinary health care teams with patients and families smoothed the transfer process. **Results**: Between 2019 and 2024, 94 IEM patients were successfully transferred. Diagnoses included intermediary metabolism defects (71.23%), lipid metabolism and transport disorders (4.25%), heterocyclic compound metabolism (2.12%), complex molecules and organelle dysfunction (6.37%), cofactor and mineral metabolism (2.12%), signalling defects (5.31%), and unclassified cases (8.51% of rare disorders, maybe non-IEM). Transition formats included 21 in-person joint visits in HSJD, 37 remote transitions during the COVID-19 pandemic, and 36 streamlined transfers via standardised protocols. Sessions, trainings, and meetings allowed the exchange of patients’ needs and protocols. **Conclusions**: The successful transference of IEM patients requires structured programmes with interdisciplinary paediatric and adult teams, joining efforts with the patient, families, and caregivers. Communication between paediatric and adult transition units is essential to promote continuity of care and patient empowerment. While constantly updated, this model has proven effective, gaining positive evaluations from healthcare professionals and patients alike, representing a scalable framework for lifelong management of IEM in adult care settings.

## 1. Introduction

Rare diseases (RDs) are a public health priority, including over 6000–8000 unique RDs globally. The European Union Regulation on orphan medicinal products defined RDs as affecting less than 50 per 100,000 individuals, or roughly 1 in 2000 individuals [1].

Almost all RD (80%) have a genetic origin and are frequently chronic and life-threatening disorders [2]. Among them, inborn errors of metabolism (IEM) include approximately 1629 genetic diseases, each defined by biochemical defects due to aberrant protein structure or function. Although singular forms of IEM are uncommon, their aggregate prevalence is notable, ranging from 1:800 to 1:2500 births worldwide. Globally, about 30 million people are affected by an RD in the European Union, and up to 3 million in Spain [3].

In 2019, the classification of IEM was expanded [4]. It was divided into six main types (24 categories and 124 disease groups), based on the affected cellular pathway: inborn errors of intermediary metabolism (nutrients, energy, or others), metabolic cell signalling, cofactor and mineral metabolism, complex molecules and organelle metabolism, metabolism of heterocyclic compounds, and lipid metabolism and transport [5]. All these IEMs involve multiple organs and require lifelong, highly specialised, and coordinated care [6].

The field of IEM has experienced substantial progress. Newborn screening (NBS) is a critical part of preventive medicine and is recognised as one of the most valuable measures to reduce morbidity and mortality during the newborn period [7,8,9,10,11]. New IEMs have also been described, and their pathophysiological bases and implications in disease are now better understood. With the advent of new metabolomics, lipidomics, proteomics, and genomics techniques, advances in diagnosis and therapeutics have multiplied. Emerging drugs and clinical trials are becoming increasingly prevalent, setting the path for new paradigms in IEM, where the specialty of metabolic medicine is consolidating [12].

Until a few years ago, most of these pathologies were lethal or disabling. Nowadays, thanks to early diagnosis and the increasing number of available treatments, 90% of IEM patients survive beyond the age of 20 [13,14]. Most of them are challenging to diagnose, and although their treatment can be complex, it is crucial to delay or stop the disease progression and limit its impact on patients’ daily lives (school, employment, social activities, and affective life).

The attention of patients with IEM has challenges to cover the specific needs and associated problems related to the disease, including those listed in Table 1 [15].

Among them, due to longer life expectancy, a new paradigm arises. Sometimes it is difficult for patients and caregivers to find reliable information about the disorder and be followed by experienced adult specialists. Specific programmes are required to ensure the transfer of paediatric patients into the adult centre when they reach adulthood. A new stream of IEM patients, successfully managed in paediatric centres, is reaching adulthood and needs to enter the adult healthcare system [16]. That new population is promoting the development of specialised care units in adult centres, and the creation of transition units between paediatric and adult centres, that guarantee proper life-long follow-up, specialised care, and functional management [17].

The concept of transition was first defined by Blum in 1993 as the “purposeful and planned movement of adolescents and young adults with chronic physical and medical conditions from child-centred to adult-oriented health care systems” [18]. Unlike the transfer, which is the specific event of moving the patient from one institution to another [17,19,20], transition represents a more dynamic, complex, and planned process. It involves paediatric and adult professionals, the patient, and his/her family or caregivers.

The transition must always be adequately developed, understood, and psychologically appropriate for the patient [21]. Its objective is to improve autonomy (transition timeline) and education about IEM disease and to offer quality care continuity [21,22,23,24]. The stages of the process should encompass a transition-focused care in paediatric outpatient clinics and inpatient settings, transfer preparation to adult care, and transition efforts within the adult care system [25]. However, the transfer process from paediatric to adult health care is still a health challenge in most settings, due to the lack of experience and established protocols. This process is especially critical for RD, including IEM [26,27,28,29,30,31], due to the need for specialised knowledge of attending professionals and the particularly vulnerable population considered.

Most adult centres have professionals specialised in RD and IEM, but regardless of paediatric centres, they are rarely constituted as units, and to our knowledge, only a few have transition and transfer programmes for IEM. This creates mistrust between patients and their families, hampering the transfer process and ultimately diminishing the success of the transition [25,32,33,34]. Even centres that have specialised adult units also generate mistrust because the approach to care, the objective, and the care criteria for monitoring an adult are different from paediatric ones. Hence, the transition team needs to progressively adapt the patient from the paediatric to adult settings and adapt new regulations, for example, ICU (intensive care unit) admission criteria, or cover new topics, including sexual and reproductive health, which must be addressed.

Failures in the transition process are associated with poor clinical outcomes, low patient/family satisfaction, increased healthcare costs, and a higher risk of follow-up loss. This scenario can have dramatic consequences in terms of morbidity and mortality [35]. On the contrary, the presence of experienced staff dedicated to transition, a transition coordinator, and specific metabolic training for adult physicians is fundamental to guarantee a successful transition and the management of IEM patients.

Recently, a consensus document was created [36] for the transition of IEM patients from paediatric to adult care, recommending planned and organised transference from a child to an adult-oriented healthcare system. We add to this concept and, from the clinical experience, include the value of creating the Transition Area, an interdisciplinary unit to better conduct the transition process. The development of the transition concept is in line with the value-based healthcare paradigm previously described by M.E. Porter [37], which evaluates the efficiency of the health service in terms of the effectiveness of interventions rather than in economic terms. Hereby, we present a functional structure transition and transfer process for IEM patients, developed collaboratively by Hospital Sant Joan de Déu (HSJD, paediatric) and the Hospital Clinic of Barcelona (HCB, adults) within the framework of the A10! programme paradigm. A10! stands for adéu, the Catalan word for ‘goodbye’, derived from the combination of ‘A’ and ‘deu’ (the number 10 in Catalan). This paper aims to share our experience in the care and transfer process to the adult hospital, integrated within the A10! program, highlighting the importance of establishing direct communication between paediatric and adult healthcare teams even before the patient transfer begins.

## 2. Materials and Methods

### 2.1. Transition and Transference Process

The transition process involves comprehensive preparation and support for patients and their families in the paediatric hospital as they approach the move to adult care. This phase employs tools, training programmes, and other initiatives aimed at equipping patients and families for the change. **It** refers to the entire long-term process that begins years before the patient reaches adulthood. It includes clinical preparation, psychosocial assessment, empowerment activities, education on self-management, and structured work with families. This process may start at 13–14 years of age, depending on the complexity of the condition.

The transfer process involves specifically the actual transference of the patient to the adult hospital, and all the subsequent hosting, as the second strategic phase of the transition. This stage involves direct collaboration between paediatric and adult care teams, where strategies such as clinical sessions and joint visits are critical to ensuring a smooth and seamless transition, which is the focus of this publication. Transference is not considered a single event. Instead, it is a sub-process within transition that requires the progressive involvement of adult-care colleagues. It includes planning meetings, information exchange, joint visits, and alignment of expectations between teams. The process of transference becomes especially critical when it involves coordination between two separate hospital systems, as it was in our case. In the transference phase, the continuity of specialised treatments and follow-up is particularly relevant and critical for metabolic disorders, which must be guaranteed across institutions.

### 2.2. The A10! Programme

The A10! is a transition programme created in HSJD to support patients with chronic conditions and their families, ensuring holistic preparation for adulthood and seamless coordination with the adult care system, ensuring continuity of care. This paper aims to share our experience in the care transfer process of IEM to the adult hospital, integrated within the A10! program, highlighting the importance of establishing direct communication between paediatric and adult healthcare teams even before the patient transfer begins.

The A10! The programme was developed in 2011 under the umbrella of Quality and Patient Experience Management HSJD, with an in-depth exploration of the needs and challenges associated with transitioning to adult care. This included interviews with patients and families, focus groups, and collaboration with professionals experienced in transition processes, particularly in fields related to common chronic conditions, such as diabetes and rheumatic diseases. During 2012–2015, a multi-disciplinary team was developed to set the transition process. By 2015, an internal protocol entitled “A10!” was published in HSJD, serving as the initial framework for transition practices. In 2018, the program expanded to address the unique needs of patients with highly complex RD and those requiring palliative care, incorporating a tailored and multidisciplinary approach. In the following years, the transition model implemented changes and improvements thanks to the participation of adolescent patients in co-creation workshops.

The communication and transition of patients with different pathologies between the hospital centres have been present for years. The first transferred patients with IEM between HSJD and HCB started in 2015. However, it was not until 2019, when the official transition of IEM patients was launched under the A10! Programme by recognised reference units for metabolic patients at the regional level (XUEC: Xarxes d’Unitats d’Expertesa Clínica), national level (CSUR: Centros, Servicios y Unidades de Referencia del Sistema Nacional de Salud), and European level (MetabERN: European Reference Network for Rare Hereditary Metabolic Disorders). By 2020, a dedicated Transition Area was established within the Quality and Patient Experience Department to support healthcare professionals, foster new alliances, and create agreements with the adult healthcare system to ensure comprehensive and sustainable transition pathways.

### 2.3. Structure of the A10! Programme

**Figure 1:**
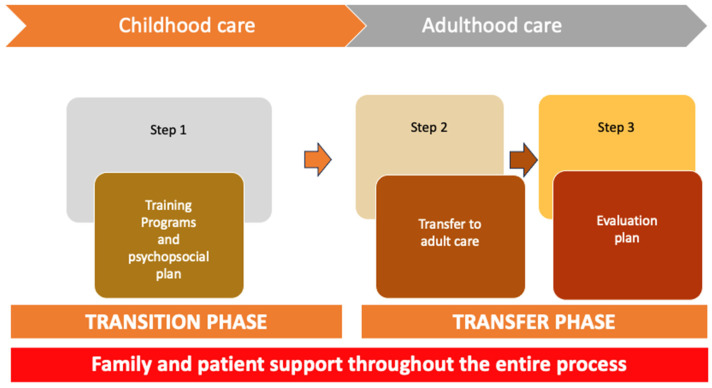
The A10! The programme is divided into two phases, the transition and the transfer phase, which in turn are divided into three stages: training and psychosocial plan (during the childhood care), and transfer to adult care and evaluation plan (during the adulthood care).

The A10! programme, summarised in Figure 1, is structured around two key stages to ensure a seamless transition for patients and their families:

**A.**  **Transition phase: preparation of patients and families**

This stage focuses on equipping patients and their families to navigate the transition process effectively. For autonomous patients, the emphasis is on fostering self-management skills, educating them about the adult healthcare system, and addressing emotional and practical concerns. For patients who are not fully autonomous, the focus shifts to supporting caregivers, ensuring they have the necessary knowledge and resources to advocate for and manage the patient’s needs. Psychoeducational support is provided to address the unique challenges faced by both groups, and readiness assessments are conducted to tailor guidance based on individual circumstances.

**B.**  **Transference phase: coordination with the adult system**

This stage involves close collaboration between paediatric and adult healthcare teams to facilitate a smooth transfer, ensuring continuity of care for all patients, regardless of their autonomy. A comprehensive transition plan is developed, including the identification of an appropriate adult care provider and the organisation of joint consultations or handover meetings. For patients who are not autonomous, this coordination also includes ensuring the adult care team is fully briefed on the patient’s dependency and caregiver support requirements. Communication between all stakeholders—patients, caregivers, paediatric teams, and adult teams—is prioritised to avoid gaps in care and to establish a strong foundation in the adult healthcare system.

This two-stage approach is designed to address the diverse needs of patients, promoting a personalised, patient- and family-centred transition that maintains continuity and quality of care.

### 2.4. Organisation, Members, and Stages of the Programme

Different stakeholders take part in this model (A–C).

**Figure 2:**
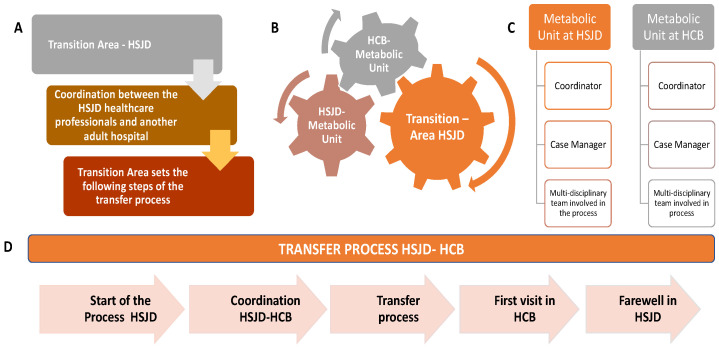
Description of the transition unit and transfer model between the paediatric hospital (HSJD) and the adult centre (HCB) for the transference of patients with inborn errors of metabolism. (**A**) The Transition Area coordinates and outlines the steps for transitioning from paediatric to adult hospital care; (**B**) The Transition Area is the gear between the two metabolic units (HSJD-HCB); (**C**) Metabolic Units are formed by interdisciplinary paediatric and adult teams, where the Case Manager (CM) connects all the professionals involved in the process (internal medicine, paediatricians, neurologists, rehabilitators, endocrine, geneticists, pharmacists, biochemists, nutritionists, social workers, psychologists, and psychiatrists, among others); (**D**) Schematic representation of the principal steps followed in the transfer process.

**A. The Transition Area** is the cornerstone of the A10 transition model, bridging HSJD and adult care teams. A schematic description of the HSJD Transition Area tasks and the transfer process between the HSJD and the HCB is shown in Figure 2.

Unlike traditional transition units, the programme adopts a team-based approach where all professionals are actively involved, creating a shared culture of transition care. This collaborative strategy ensures a patient-centred focus, with staff trained to understand and implement key transition goals.

Developed between 2020 and 2021, the Transition Area is staffed by multidisciplinary HSJD teams consisting of a transition coordinator, a specialist, case managers (often nurses), training programme representatives, admissions officers, and RD representatives. Case managers play a critical role, providing specialised support throughout the process. By establishing agreements with adult hospitals and fostering collaboration, the Transition Area ensures continuity of care while leveraging its network as a long-term strategy for HSJD. This Transition Area in HSJD is also responsible for the coordination between the paediatric and the adult centres for contact with the Metabolic Units. It provides support during the transition process by promoting agreements with an adult hospital and guaranteeing continuity of care for patients.

**B. Metabolic unit structure**. The HSJD and HCB Metabolic Units are formed by a coordinator, a case manager, and an interdisciplinary team in the paediatric and adult centre, coordinated by the Transition Area. Paediatric and adult metabolic units are integrated by paediatricians, internal medicine specialists, neurologists, rehabilitation specialists, endocrinologists, pharmacists, biochemists, dietitian–nutritionists, social workers, psychologists, psychiatrists, and genetic counsellors, among others.

**C. Stages of the transition and transference between HSJD—HCB**. Transition is a dynamic process in which some phases may need to be revisited or extended; however, it was structured into the following steps:

**(i) HSJD preparation**: The first step of this process consisted of detecting those patients ready to start the transition pathway. Patients must be 13–14 years old and affected by an IEM. A consensus is established between the patient/family and the reference healthcare team. The patients’ and families’ training and empowerment are evaluated, and the transfer to the adult hospital is initiated at the age of 16–17, to be finally achieved at 18 years old.

**(ii) HSJD–HCB Coordination**: Coordination between the two centres is carried out through clinical meetings aimed at reviewing the information of all patients ready to be transferred. This information includes the follow-up of patients’ registries and their clinical evolution. The HSJD case manager uploads a generic and transversal clinical summary report to the HCB digital platform involving all medical aspects (treatments, or medical equipment). Social work and psychology/psychiatry reports are also attached. Finally, a detailed referral request document, which includes a follow-up proposal from the different services involved, and if there is a previous link with the centre, is uploaded. Different protocols have been agreed upon for the follow-up of patients with various diseases. An essential part of the transfer process is to ensure that patients will continue receiving their specific pharmacological and dietary treatment throughout the transition and once they arrive at the adult hospital. In our case, during the pre-transfer coordination meetings between the two centres, HSJD notifies whether patients are on specialised treatments, so that HCB can initiate the necessary administrative procedures required by health authorities to maintain their continuity and funding. Furthermore, it has been agreed to allow patients to continue collecting their medication at HSJD for a time-limited bridging period after their transfer to HCB, when necessary, to prevent any interruption in treatment in case of administrative delays.

The transfer of medical information is carried out through a shared platform between both units, in full compliance with Spanish Organic Law 3/2018 on the Protection of Personal Data and Guarantee of Digital Rights, as well as with the European General Data Protection Regulation (EU 2016/679) [38], ensuring maximum security. The HCB case manager receives the transfer request and coordinates with the specialist(s) at the HSJD to agree on joint visit scheduling. The two case managers are the key pieces of this coordination.

In addition, in the case of IEM transitions, both Metabolic Units have term meetings (currently a quarterly meeting) where clinical cases, new diagnoses, clinical trials, and problems with transitions are discussed. Moreover, discoveries about disease diagnoses or treatments are shared in these meetings. The internships with medical adult staff in the paediatric centre supported the understanding of previous patient management before starting the programme. It is extremely important to describe the number of patients’ decompensations, their cause, and treatment.

Additionally, scientific training and sessions for professionals and patients in both centres (online and face-to-face) are held. An example of this information exchange is the annual conference for RD held in the HCB, where all these topics are discussed by specialised professionals, students, stakeholders, patients, and caregivers.

**(iii) Pre-transition visit at HSJD**: This phase is necessary to prepare the transition plan and process the patient’s information. The Transition Plan includes a medical and emergency plan (nursing, social work, or psychiatric/psychological support) and a psychosocial plan (need for other community resources and supports). The content of the reports is explained to the patient/family, and any doubts are resolved. The complete information is then uploaded to a digital platform that permits the sharing of patients’ clinical records among different hospitals in Catalonia. In this phase, the patient is introduced to the HCB referent, who will follow him/her in the future. The HCB medical referent is present in the last visits (at least one), either in person or online.

The main objective is to consolidate adequate communication between the specialists and the patient/family, supporting the HCB team who will receive the patient. It is essential to help with any queries in the initial stage of patient care to ensure a good reception at the receiving centre.

**(iv) First visit to HCB**: The patient is seen at the adult hospital (HCB) by the referring physician they had previously met at the paediatric hospital (HSJD). In some cases, the visit of the HSJD medical staff has also been required at HCB. It is important to have the same professional meet in HSJD, for the trust of the family and the patient.

**(v) Transition completed and post-transfer evaluation**: Once the transfer to the adult hospital is completed, the HSJD coordinator and the specialist set a new appointment with the patient/family 6 months later. This meeting can be either telematic or in person and aims to facilitate adaptation to the new adult environment. Compliance with the visit schedule is evaluated. This phase is necessary to ensure that all medical and psychosocial needs are covered to complete the transition process. Follow-up and monitoring of the transition is needed to guarantee the continuity of care and detect any deviations from the protocol or unforeseen difficulties.

## 3. Results

The A10! programme to transfer IEM patients from HSJD to the HCB started with cases with less complex clinical care needs (i.e., patients with phenylketonuria [PKU] under stable metabolic control) and gained structure and complexity over time.

The standardisation of the process required the implementation of different tracks and circuits (Figure 2). The training programme was deployed for education and training plans for both patients and families. The ages of 13 and 14 years were considered appropriate for initiating patient training aimed at promoting autonomy in disease management, although adult supervision—typically by family members—remains necessary. Patients between 16 and 18 years, patients were assessed for their ability to assume responsibility independently and autonomously. At this stage, their knowledge and disease management skills were evaluated, with reinforcement of competencies already acquired and emphasis on those still requiring development. In cases where the patient presents with intellectual disability, continuous involvement of family members remains essential.

The Psychosocial plan was implemented to accompany and advise about changes in the adult stage. This step aims to help patients cope with the changes due to the disease. They will learn how to manage the conditioning factors in their lives regarding basic activities of daily living and know about some important social aspects, such as changes related to social benefits, labour, education, etc. The transfer to the adult care system was established after specific communication of patients’ characteristics and needs with the adult hospital, for continuity of care, and the safety in the transfer process. The evaluation plan was established for monitoring patients’ needs once transferred into the adult hospital. Specifically, 6 months after the transference, HSJD sends a survey to patients and their families to evaluate the transition process. Similarly, in the HCB, focal groups and patients’ experience have been created according to the Patient Experience Observatory from the HCB to evaluate strengths and weaknesses of the process.

Additionally, in the adult centre, novel circuits needed to be implemented to standardise this model, including the development of protocols for each disease or group of diseases, as well as emergency and decompensation protocols.

Following those measures, from November 2019 to July 2024, 94 patients with IEM were transferred between HSJD and HCB metabolic teams, guided by the Transition Area.

Briefly, Table 2 shows the classification of transferred IEM patients according to international guidelines, detailing the percentages of disease depending on the affected metabolic pathway: intermediary metabolism defects (71.23%), lipid metabolism and transport (4.25%), metabolism of heterocyclic compounds (2.12%), complex molecules and organelle metabolism (6.37%), metabolism of cofactors and minerals (2.12%), cellular metabolic signalling (5.31%) and rare disorders maybe non-IEM (8.51% with non-definite molecular diagnosis).

The specific diagnosis and epidemiology of transferred patients are also described in Table 3.

The age of patients at transference ranged from 17 to 45 years (mean age 20.3 years), of whom 40 were female (42.5%), and 54 were male (57.4%). Of note, the 45-year-old patient was an asymptomatic mother, secondarily diagnosed on account of genetic familial counselling, after her newborn was diagnosed.

When considered in detail, most of the patients were affected by PKU, accounting for 21.28% (n = 20) of the cohort. Other identified inborn errors of metabolism (IEMs) included glutaric aciduria type I (4.26%, n = 4), cerebral creatine transporter deficiency (2.13%, n = 2), non-PKU hyperphenylalaninemia (3.19%, n = 3), classic galactosemia (2.13%, n = 2), neuronal glucose transporter deficiency (4.26%, n = 4), mitochondrial diseases (6.38%, n = 6), glycosylation defects (3.19%, n = 3), other organic acidurias (3.19%, n = 3), and hereditary fructose intolerance (5.32%, n = 5). A substantial proportion of patients (40.42%, n = 38) presented other individual IEMs or non-molecularly classified diseases.

For the transference of these 94 patients, 21 required a face-to-face visit with the adult team at the paediatric centre, 37 required a telematic visit (due to the SARS-CoV-2 pandemic), and 36 patients did not require an initial joint visit thanks to protocol standardisation.

At present, all of them have been successfully transferred and are regularly followed up on in the HCB. Now, two patients are starting the transition process, one patient temporarily left the country, two patients moved to different hospitals, and four patients did not show up at the scheduled visits and had to be rescheduled. Due to the SARS-CoV-2 pandemic, some patients temporarily lose their follow-up. Not only the transference, but also relevant efforts should be focused on patient follow-up and maintenance.

All therapeutic options were carefully supervised and optimised in the adult centre, although most patients continued the treatment plan proposed by the paediatric team. Patients requested the same resources they had in the paediatric centre, including psychologists, physiotherapists, neurologists, dietitian–nutritionists, and gastroenterologists. The interdisciplinary team is hereby required to be implemented, both in the paediatric and adult hospitals.

Both multidisciplinary teams have discussed each of the transferred patients at quarterly meetings. Different protocols have been agreed upon for the follow-up of patients with various diseases, including emergency protocols. Also, a protocol to communicate critical laboratory results has been implemented to cover potential imbalances in the follow-up of patients. In these cases, communication between case managers of both teams is crucial, and for each of these patients, a meeting or communication between both parties has been established.

The transfer of paediatric patients with IEM is a complex process, and we herein reflect on the main problems that emerged, shown in Table 4, which summarises the difficulties and needs for a successful transition programme.

Also, given our experience and based on this model of transition, we have considered making some recommendations (Table 5).

The key points of the process have been the possibility of conducting joint visits and consultations, the implementation of monthly clinical sessions among professionals within each unit, and the establishment of quarterly sessions between both teams and hospitals. These sessions were focused on planning the transfer of patients and discussing those who had already been transferred. Furthermore, this system has enabled the development of studies thanks to the traceability of the process and participation in research aimed at improving diagnostic and therapeutic workflows. One of the programme’s strengths is that it provides families with support and guidance regarding the changes that occur during the transition to the adult system, including social benefits, special education, and residential services.

The new team was introduced to patients and family members, who were also invited to participate in sessions addressing disease-specific and treatment-related topics, as well as new areas such as sexuality and reproductive genetic counselling. Clinical consultations should progressively incorporate elements of health education, the cultivation of self-management competencies, and the exploration of critical topics such as treatment adherence, mental health and reproductive planning.

## 4. Discussion

This experience shows that the transition of IEM patients from paediatric to adult hospitals requires time and effort. We believe that it is necessary to transform the “one-day process” into one that contemplates different meetings over a more extended period, which includes systematic and consensual strategies in the care of adolescents, and that understands the transition based on the concept of “interdependence”.

One of the main achievements of this experience was to reach a consensus and systematise a transition process for ÌEM patients and empower the adult centres in some specific, until now, paediatric diseases.

Each year, hundreds of adolescents with chronic or complex conditions transition from paediatric care to adult-focused centres or services, ensuring continuity of care and disease management. For patients and families, this change is a process of training and empowerment that frequently generates doubts, concerns, and fears.

Evidence suggests that patient empowerment is a critical part of effective chronic disease management reform, as it will help maximise efficiency and value in healthcare systems [41]. This is not achieved in one day. Our approach offers the required time to get adapted to the transition into the adult hospital, providing emotional and social-work support, and a safe context for treatment adherence monitoring and disease follow-up. Creating recommendations for improving treatment adherence during transfer or multidisciplinary follow-up for these patients is important [39].

Challenges in transition are particularly striking for patients with IEM, since leaving their paediatric specialist can overwhelm patients and their families. Thus, transitions must be carefully planned and organised from the child-centred to the adult-oriented healthcare system.

Although it is well recognised that adult patients should be treated in an adult environment with adult teams trained to manage their condition, the number of clinics offering this service is still low [16,26,42], and only a few centres have created specific transition guidelines [15,43,44,45].

At this point, we presented a successful model of transition of patients with IEM from paediatric to adult hospitals, which permitted us to transfer up to 94 patients and could easily be extended to other adult centres. The transition model is inspired by other transition models and experiences found in the literature [17,18,19,20,21,22,23,24,25,26,27,28,29,30,31,32,33,34,35,36], bringing improvements, such as the Transition Area.

Thanks to the feedback of the transition and transference process provided by patients and families through established surveys, and the collaboration with a team of professionals from the Department of Quality and Patient Experience of the HSJD, a guide was created, available on the web page of the hospital ‘transition to Adult’s Hospital’ [46] to answer the main questions and concerns that these adolescents may have throughout the transition process. The guide is the first of these characteristics that exists in Spain. This document, prepared with the collaboration of patients, families, and professionals, provides adolescents and their families with the information they need in each phase of the transition process, advises them on issues that they have identified as important, and includes a self-assessment form. The guide is freely accessible and can be consulted online [46]. Additionally, the transfer process described herein should also be subjected to regular evaluation surveys by patients and families. This is a dynamic system with constant improvement that benefits from feedback given by patients and families, either personally or through questionnaires. The present transition model also evaluates patient and caregiver experience, aiming to reduce weaknesses and improve the strengths of this programme.

The paediatric team recommended starting the transition process with patients around 13–14 years old, to be finally transferred at 18. However, age should not be considered the unique criterion for transfer. Clinical stability is critical for transition; patients must be in proper metabolic control and treatment adherence. In addition, the affective and emotional aspects of the patient and his or her family are equally relevant. The transfer process must start at a time of disease stability, in the absence of major therapeutic changes, and when the patient and their family are ready to be transferred.

Introducing the new team to patients and families is essential to achieving this goal. It facilitates accessibility and the establishment of protocols for communication and contact with patients. Providing information about the new care pathways is essential, but also empowering the patient and recognising their novel role in the management of the disease. In this regard, training sessions for professionals, but also patients, families, and patient associations, have been essential.

Another cornerstone to implement the transition programme is the transfer of information from the paediatric to the adult hospital. Information should always precede patient transference. For instance, before involving the patient in any referral process, patient information needs to reach the receiving team, and only after positive feedback is given can the process move forward. Communication is essential between centres, coordination is crucial to succeeding, and information must be constantly provided to the patient and their family along the different steps of the process.

Although the focus of this study is on the clinical and organisational aspects of transition, it is important to recognise that the continuity of ultra-expensive therapies for patients with inherited metabolic disorders also depends on broader system-level financing mechanisms. Across many European health systems, the long-term funding and resource allocation required for these treatments represent an ongoing structural challenge that extends beyond the remit of individual hospitals. Ensuring therapeutic continuity, therefore, requires transparent, coordinated policies that support equitable access throughout the patient’s lifespan. Within this context, transition programmes play a complementary role by anticipating organisational needs, coordinating with adult centres, and addressing potential administrative or financial barriers early, so that no interruption in treatment occurs during the transfer process.

A key aspect of the process is that the transition process must be managed by expert clinical units and professionals with relevant expertise in IEM. These expert units should oversee clinical care, draw up clinical guidelines for diagnosis, manage treatment options, patient follow-up, adapt pharmacy services, create patient registries, establish relationships with patient associations and other national or international adult teams, and maintain proper research activity to foster advances that cover patients’ needs. In the case of the adult IEM unit, we set the coordination with a physician specialist in Internal Medicine, who can lead multidisciplinary teams and elaborate personalised management plans for each patient [47]. However, the specific requirements of each centre and transition unit should be considered.

In general, transition processes lack specific consensus. The literature in this regard is very heterogeneous, and the conclusions are based on expert opinions and recommendations [40,48,49,50,51,52]. An important aspect should be to define the quality criteria of a transition unit and subsequently conduct research to assess which models most effectively achieve these objectives and simultaneously add value [40]. It is difficult to establish a single, validated, and reproducible model in rare diseases, with few transitions, and adapted models are needed [48].

Undoubtedly, the success of the herein presented model is the creation of a Transition Unit, a network of organised professionals working together to coordinate the paediatric and the adult centre, instead of disorganised specialists working autonomously. This unit guarantees the most accurate management of patients and guides patients and their families so that the young patient becomes an independent adult who actively participates in his/her health care.

The usefulness of transition units is assumed by most specialists, but it is necessary to integrate them into our hospital routines and settings. There is an urgent need to invest resources and staff in creating or strengthening these units, as well as in understanding new protocols and tools to improve their function.

The challenge for research in this area is to understand these units as an interdisciplinary process; therefore, it is vital to integrate all parties in their development or their improvement [49].

On the contrary, several barriers could prevent a successful transition [43]. Among them, the lack of coordination between the paediatric and adult care teams, anxiety over expectations in the adult care system, complex clinical needs, inappropriate preparation for the adult care system, less accessible adult system, less accessibility to other medical specialties, lack of time for healthcare professionals, or lack of psychological support.

Despite the improvements brought by the implementation of this programme, other needs remain unmet, such as establishing a registry of decompensations for each patient or disease, standardising the evaluation of patient experience, creating pathways to provide emotional support to patients and families, implementing shared decision-making and life expectation management, as well as sharing the experience with other centres (which motivated this manuscript).

In addition, it is essential to be able to evaluate the satisfaction generated by the transition process and the assessment of the follow-up of the transferred patient, and the most significant challenge will probably be to achieve a more inter-institutional strategy, which can be maintained over time as part of the healthcare system.

We encourage other centres to incorporate the active participation of patients and their families in the shared decision-making process, facilitating their acceptance of the new expert adult multi-disciplinary team.

## 5. Conclusions

The success of healthcare transition processes hinges upon early-stage planning, the patient’s active and gradually increasing engagement in disease self-management, and effective coordination between paediatric and adult care teams. To guarantee proper follow-up of RD patients, especially those with IEM, it is necessary to develop lifelong, specialised care and functional transition services from paediatric to adult care. Some conclusions arise from our experience. First, we can confidently assert that the “A10! Program” is an effective model, since patients, families, and medical professionals express satisfaction with this process. Second, to foresee that the role of the Transition area and the coordinated metabolic units of both centres is essential to succeed. Third, while the role of the internal medicine physician is critical in adult centres, the participation from the rest of the interdisciplinary team is fundamental to conducting a proper and integral management of the patient. Finally, it is highly recommended to encourage the involvement of patients and families in the process to guarantee a smooth and seamless transition. In parallel, some concerns arise. The most important limitation may be the potential lack of specialised staff in IEM in adult hospitals or even the commitment of adult hospital centres to transitions. These job positions and units should be created. Similarly, training of adult physicians in paediatric hospitals should be implemented, not only for primary care professionals, but also for internal medicine specialists, endocrinologists, or neurologists. In addition, teams are usually overwhelmed with heavy daily workloads, and the organisation of the transfer process is usually done during extra working hours. For this reason, the transition programmes must be revised, updated, and recognised among the professionals and the hospital centres in a dynamic process for constant improvement, where stakeholders must provide resources and staff to create or strengthen IEM transition units.

## Figures and Tables

**Table 1:** Challenges and needs of rare diseases.

Problems of Rare Diseases	Needs of Rare Disease
The aetiology and diagnosis are complex	They need more specialised genetic and biochemical studies
Unknown to society and professionals	It is necessary to spread more information in society and among professionals
In general, they are hereditary diseases and will usually begin at an early paediatric age	They require trained paediatric, but also adult physicians
They are chronic and progressive diseases	They require special care, trained adult physicians, rehabilitation, and family support needs
High morbidity and high levels of dependence	They need advanced social work
Little financial support for the Health System and a lack of specific therapies	They need multi-disciplinary support and industry interest
Rare diseases often require personalised medicine due to their unique nature and variability	It is important to promote research in precision medicine, which allows for the identification of the specific genetic and biological characteristics of each patient
The increase in new diseases and cases of rare diseases represents a significant challenge	Promote the creation of specialised centres and multidisciplinary teams. Collaboration between institutions, and access to genomic data.

**Table 2:** Classification of transferred IEM patients according to the international classification of hereditary metabolic diseases.

Affected Metabolic Pathway	Categories	N	%
Intermediary metabolism: nutrients	1. Amino acid metabolism disorders	39	41.48
	2. Peptide and amine metabolism disorders	0	0
	3. Carbohydrate metabolism disorders	12	12.76
	4. Fatty acid and ketone body metabolism disorders	2	2.12
Intermediary metabolism: energy	5. Energy substrate metabolism disorders	7	7.44
	6. Mitochondrial DNA-related disorders	4	4.25
	7. Nuclear-encoded oxidative phosphorylation disorders	1	1.06
	8. Mitochondrial cofactor biosynthesis disorders	0	0
	9. Mitochondrial DNA maintenance and replication disorders	0	0
	10. Mitochondrial gene expression disorders	1	1.06
	11. Other mitochondrial function disorders	1	1.06
	12. Metabolite repair/correction disorders	0	0
13. Miscellaneous intermediary metabolism disorders		0	0
Lipid metabolism and transport	14. Metabolite metabolism disorders of lipids	4	4.25
	15. Lipoprotein metabolism disorders	0	0
Metabolism of heterocyclic compounds	16. Nucleic acid, Nucleotide, and nucleic acid metabolism disorders	2	2.12
	17. Tetrapyrrole metabolism disorders	0	0
Complex molecules and organelle metabolism	18. Inborn errors of glycosylation	1	1.06
	19. Organelle biogenesis, Dynamics, and interaction disorders	1	1.06
	20. Complex molecule degradation disorders	4	4.25
Metabolism of cofactors and minerals	21. Trace element and metal disorders	2	2.12
	22. Vitamin and cofactor disorders	0	0
Cellular metabolic signallers	23. Neurotransmitter disorders	4	4.25
	24. Endocrine metabolic disorder	1	1.06
Total of molecularly classified IEM		86	91.48
Non-molecularly classified disorders according to ICIMD		8	8.51
Total		94	

**Table 3:** Specific diagnosis and gender of transferred patients.

Diagnosis of IEM	N	Male	Female
Phenylketonuria	20	11	9
Mitochondrial diseases	6	1	5
Hereditary fructose intolerance	5	3	2
Classic galactosemia	4	3	1
Glutaric aciduria type 1	4	3	1
Neuronal glucose transporter deficiency	4	1	3
Hyperphenylalaninemia	3		3
Defects of Glycosylation	3	3	
Other organic acidurias	3	1	2
Cerebral creatine transporter deficiency	2	2	
Carbamoyl phosphate synthetase 1 deficiency	2	2	
Encephalopathy no filiated	2	2	
Succinate dehydrogenase deficiency	1		1
3-phosphoglycerate dehydrogenase deficit	1		1
Cerebral folate transport deficiency	1	1	
Carnitine palmitoyltransferase 2 deficiency	1		1
Rhizomelic chondrodysplasia punctacta	1	1	
Ceroid lipofuscinosis neuronal type 3	1		1
Ceroid lipofuscinosis neuronal type 2	1	1	
Non-progressive congenital ataxia	1	1	
Methylmalonic aciduria and homocystinuria, cblC type (cblC deficiency)	1	1	
Argininosuccinic aciduria	1	1	
Mucopolysaccharidosis type 3	2	1	1
3-Methylcrotonylglycinuria	1		1
Ornithine transcarbamylase deficiency	2	1	1
Hyperornithinemia–Hyperammonemia–Homocitrullinuria syndrome (HHH syndrome)	1	1	
OPA1 (*optic atrophy 1*) deficiency	1		1
Mitochondrial serine hydroxymethyltransferase deficiency	1	1	
Tyrosinemia type 1	1	1	
Aminoacylase 1 deficiency	1	1	
Cerebrotendinous xanthomatosis	1	1	
L-2-Hydroxyglutaric aciduria	1		1
Lesch-Nyhan Syndrome	1	1	
Phosphomannomutase 2 deficiency	1	1	
Leber hereditary optic neuropathy	1	1	
STX1A-related Syntaxin 1A deficiency	1		1
Classical homocystinuria	1		1
Long-chain acyl-CoA dehydrogenase deficiency	1	1	
**Non-Molecularly Classified Disorders According to ICIMD**			
Neurodevelopmental encephalopathy	3	1	2
Deep global encephalopathy	1	1	
Non-progressive encephalopathy	1	1	
Multicystic encephalopathy	1	1	
Spastic tetraparesis	1		1
Sodium Voltage-Gated Channel Alpha Subunit 4 (SCN4A) mutations	1	1	
**Total**	**94**	**54**	**40**

**Table 4:** Difficulties and needs for a successful transition programme.

Main Difficulties for the Transition Process	Main Needs for a Transition Process
No financial support for these programmes	Time and staff dedicated to transition in both adult and paediatric hospitals
Lack of metabolic staff (paediatricians and adult physicians including neurologists) endocrinologists, dietitians. nurses…)	Transition protocols in adult and paediatric hospitals
No official metabolic position both in adult and paediatric hospitals	Metabolic paediatricians and metabolic adult physicians
Lack of manager of cases (specialised nursing)	Metabolic training, mainly for adult staff
Lack of time or unaccounted time in the working day to dedicate to the transition	Psychologists and social workers trained in these diseases

**Table 5:** The recommendations for a successful transition.

For Patient/Family/Carer	For Professionals	For Healthcare Organizations
Inform and empower the patient, family, or caregivers about the disease	A complete detailed paediatric report on the patient’s evolution up to the time of transition. This record must include (at least) the following information [39]: Clinical diagnosis (age at diagnosis, clinical characteristics, genetic diagnosis), relevant personal and clinical history, pharmacologic treatment: the up-to-date treatment plan, drug levels, specific therapeutic monitoring, and report on treatment adherence, adverse effects or drugs tried without success and recommendations for clinical follow-up specialists	Designate a team devoted to transitions
Guidance on healthy habits. Encourage treatment adherence and follow-up	Ensure that the pharmacy circuit is suited for the new circumstances, to keep providing the medical treatment during and after the transfer	Carry out a standardised written protocol
Information at all times during the transition process	Quality control of the transition model. including a registry of transferred patients and periodic updates of the transition protocol	Publicise the transition programme among professionals
Introduce the receiving teams	Monitor transferred patients for at least the first year	Design the transition processes through a value chain [40]
Follow a schedule	Active and effective communication between centres. Possibility of establishing progressive contact with the adult team	
Given the opportunity to express fears and reluctance during the process	Train to assess the maturity of patients during the process	
Always consider the social sphere of the patient as well as their preferences regarding the adult centre (distance may influence)	Agree on the transition between the paediatric and adult teams	
	Appoint a “coordinator” during the transition process	
	Possibility of creating a “transition visit” in which the two specialists (paediatric and adult team) care for the patient together	

## Data Availability

Data contained within the article can be shared upon request.

## Abbreviations

The following abbreviations are used in this manuscript:

HCB	Hospital Clinic of Barcelona
IEMs	Inborn errors of metabolism
HSJD	Hospital Sant Joan de Déu;
RD	Rare disease
